# Sex-related differences in the risk factors for in-hospital mortality and outcomes of ischemic stroke patients in rural areas of Taiwan

**DOI:** 10.1371/journal.pone.0185361

**Published:** 2017-09-21

**Authors:** Cheung-Ter Ong, Yi-Sin Wong, Sheng-Feng Sung, Chi-Shun Wu, Yung-Chu Hsu, Yu-Hsiang Su, Ling-Chien Hung

**Affiliations:** 1 Department of Neurology, Chia-Yi Christian Hospital, Chia-Yi, Taiwan; 2 Department of Nursing, Chung Jen Junior College of Nursing, Health Science and Management, Chia-Yi, Taiwan; 3 Department of Family Medicine, Chia-Yi Christian Hospital, Chia-Yi, Taiwan; National Yang-Ming University, TAIWAN

## Abstract

**Background and purpose:**

Sex-related differences in the clinical presentation and outcomes of stroke patients are issues that have attracted increased interest from the scientific community. The present study aimed to investigate sex-related differences in the risk factors for in-hospital mortality and outcome in ischemic stroke patients.

**Methods:**

A total of 4278 acute ischemic stroke patients admitted to a stroke unit between January 1, 2007 and December 31, 2014 were included in the study. We considered demographic characteristics, clinical characteristics, co-morbidities, and complications, among others, as factors that may affect clinical presentation and in-hospital mortality. Good and poor outcomes were defined as modified Ranking Score (mRS)≦2 and mRS>2. Neurological deterioration (ND) was defined as an increase of National Institutes of Health Stroke Score (NIHSS) ≥ 4 points. Hemorrhagic transformation (HT) was defined as signs of hemorrhage in cranial CT or MRI scans. Transtentorial herniation was defined by brain edema, as seen in cranial CT or MRI scans, associated with the onset of acute unilateral or bilateral papillary dilation, loss of reactivity to light, and decline of ≥ 2 points in the Glasgow coma scale score.

**Results:**

Of 4278 ischemic stroke patients (women 1757, 41.1%), 269 (6.3%) received thrombolytic therapy. The in hospital mortality rate was 3.35% (139/4278) [4.45% (80/1757) for women and 2.34% (59/2521) for men, *p* < 0.01]. At discharge, 41.2% (1761/4278) of the patients showed good outcomes [35.4% (622/1757) for women and 45.2% (1139/2521) for men]. Six months after stroke, 56.1% (1813/3231) showed good outcomes [47.4% (629/1328) for women and 62.2% (1184/1903) for men, *p* < 0.01]. Atrial fibrillation (AF), diabetes mellitus, stroke history, and old age were factors contributing to poor outcomes in men and women. Hypertension was associated with poor outcomes in women but not in men in comparison with patients without hypertension. Stroke severity and increased intracranial pressure were associated with increased in-hospital mortality in men and women. AF was associated with increased in-hospital mortality in women but not in men compared with patients without AF.

**Conclusion:**

The in-hospital mortality rate was not significantly different between women and men. Functional outcomes at discharge and six months after stroke were poorer in women than in men. Hypertension is an independent factor causing poorer outcomes in women than in men. AF is an independent factor affecting sex differences in hospital mortality in women.

## Introduction

Stroke is one of the leading causes of death and disability in the world. Even in developed countries, the in-hospital mortality in stroke patients is 3%–11% [1–3]. The in-hospital death rate and poor outcome rate in hemorrhage stroke patients is significant higher than in ischemic stroke patients. However, the in-hospital death rate still 4.7% and poor outcome rate is 43.8% in ischemic stroke patients[2].

Current treatments for stroke have evolved from being fairly conservative to very active [4, 5]. Despite advances in the treatment of stroke patients, however, the mortality rate of the disease remains high [5]. Identifying a patient’s risk of mortality at admission could help physicians provide valuable prognostic information to patients and their families and identify patients who are at high risk for poor outcomes and may thus require more intensive treatment [6]. Several studies have reported sex differences in the management of patients with ischemic stroke. Compared with men, women have been found to be more likely to receive, in the acute phase, less aggressive therapy and, in general, reduced diagnostic and therapeutic procedures [7–9]. Recent research has also reported racial and gender differences in stroke severity and clinical outcomes [10]. Females suffered from more severe strokes, had higher short-term mortality [7, 11, 12], and were subjected to different management techniques in comparison with males. Thus, poor outcomes in women may be related to differences in stroke management in the acute phase. However, other studies found no differences in outcomes based on sex after controlling for other factors [13–15]. A few studies revealed that female patients had lower mortality rates than men [16, 17]. While most studies included covariates in their analyses, the factors attributed to sex differences in terms of outcomes and fatality remain unknown, and further investigation of sex differences in factor affecting the outcomes of stroke patients is warranted. Because women have longer life expectancies and much higher incidence rates of stroke at an older age than men, the number of strokes in the former is higher than that in the latter [7]. As the impact of stroke is higher in women than that in men, investigating differences in strokes between men and women is necessary. In this work, we aimed to investigate sex differences in the risk factors for in-hospital fatality and the factors affected clinical outcome.

## Method

### Data collection

Data were collected during stroke registration from patients with acute ischemic stroke admitted to the stroke unit of a teaching hospital between January 1, 2007 and December 31, 2014. Our hospital is a 1000-bed acute-care teaching hospital in southern Taiwan. The hospital has a stroke unit helmed by a neurologist with expertise in strokes. The study was approved by the research ethics board of the hospital, and written informed consent was obtained from the patients or their families before data collection. Trained neurology research nurses recruited and registered the data of consecutive patients with acute stroke or transient ischemic attack based on chart reviews and patient or family interviews. All patients with acute neurological symptoms were initially attended to in the emergency department. After neurological evaluation, cranial computed tomography (CT) scan, or magnetic resonance imaging (MRI), the patients were admitted to the stroke unit, which is part of the Department of Neurology. Fasting blood sugar, cholesterol, and triglycerides were recorded on the day following admission, and neurological examinations were performed every day.

During hospitalization, stroke management was performed according to the guidelines of stroke management in Taiwan[18]. The National Institutes of Health Stroke Score (NIHSS) and the modified Rankin score (mRS) were used to evaluate stroke severity and functional outcomes, respectively. CHADS_2_ Score was used for stroke risk evaluation [19]. We evaluated functional outcomes at discharge and six months after stroke onset. Neurological deterioration (ND) was defined as an increase of ≥ 4 points in the NIHSS [20], and good and poor outcomes were defined as mRS ≤ 2 and mRS > 2, respectively. Stroke was classified as large-artery atherosclerosis (LAA), cardioembolic stroke (CE), small vessel occlusion (SVO), undetermined etiology (UN), and other determined etiology (OD) according to the Trial of Org 10172 in Acute Stroke Treatment (TOAST) criteria [21]. The five subtypes of strokes included LAA (n = 1141), SVO (n = 1485), CE (n = 604), UN (n = 1039), and OD (n = 9). Only patients with ischemic stroke were included in the current study, and patients presenting transient ischemic attack, intracerebral hemorrhage, or subarachnoid hemorrhage were excluded. Other exclusion criteria were not applied during patient selection in the present study. The following information was recorded for each patient: demographic data (age, sex, and body mass index), vascular risk factors [hypertension, diabetes mellitus, atrial fibrillation (AF), hyperlipidemia, smoking, and heart disease], stroke score (NIHSS), neuroimaging, carotid duplex, and outcome. Variables affecting stroke outcome were assessed. Age was categorized as < 65, 65–74, 75–84, or > 85 years. Hypertension was defined by a systolic blood pressure of ≥ 140 mmHg, diastolic blood pressure of > 90 mmHg, self-reported history of hypertension, or use of an antihypertensive agent. Diabetes mellitus was defined in terms of elevated fasting blood glucose over 126mg/dl or hemoglobin A1C level over 6.5%, self-reported patient history of diabetes mellitus, or regular use of anti-diabetic medications. AF was defined in terms of history or identification of AF from an electrocardiogram. Hyperlipidemia was defined in terms of total serum cholesterol levels of > 200 mg/dL or low-density lipoprotein levels of > 130 mg/dL, as measured during the acute stage of stroke. Heart disease was defined by a history of ischemic heart disease or congestive heart failure. Increased intracranial pressure (IICP) was defined by the evidence of brain edema, mass effect, or brain shift syndrome in cranial CT or MRI scans, showing association with clinical deterioration [16]. Transtentorial herniation was defined by brain edema, as seen in cranial CT or MRI scans, associated with the onset of acute unilateral or bilateral papillary dilation, loss of reactivity to light, and decline of ≥ 2 points in the Glasgow coma scale score [7]. Hemorrhagic transformation (HT) was defined as signs of hemorrhage in cranial CT or MRI scans [13]. Outcome variables included infection, cardiac or vascular events, and respiratory disease. The study was approved by the research ethics board of the hospital (CYCH-IRB: 096022) and written informed consent was obtained from the patients or their families before data collection.

### Statistical analyses

Chi-square and independent (student’s) *t*-tests were employed for univariate analysis, Mann-Whitney U test was used for CHADS2 analysis; a *p* value of < 0.05 was considered statistically significant. Logistic regression was used to identify independent risk factors of in-hospital mortality. Multivariable logistic regression was used as the independent risk factor for stroke severity, ND, HT, and stroke outcome. Analysis was performed using MedCalc statistical software version 12.3 (MedCalc Software, Ostend, Belgium).

## Results

### Background characteristics

From January 1, 2007 to December 31, 2014, 4278 stroke patients were admitted to the hospital. The characteristics of these patients are presented in Table 1. The mean age was 69.9 years (men, 68.29 years; women, 72.18 years), and 1757 (41.1%) of the patients were women. Female patients were generally older than males. Comorbid conditions and stroke severity, as defined by the NIHSS, are given in Table 1. While women were more likely than men to have diabetes, hypertension, AF, and hyperlipidemia, men were more likely than women to report a history of smoking and stroke. On admission, women presented more severe neurological deficits than men. Severe stroke (NIHSS score > 15 on admission) was more prevalent in women than in men (20.9% vs. 13.1%, *p* < 0.01), and women had a higher frequency of CE and lower frequency of LAA than men. In the patients with atrial fibrillation, the CHADS2 Score was higher in women then in men [Median 2 (2–3) in women, 2 (1–3) in men, *p* < 0.01]. During hospitalization, no sex difference was observed in terms of frequency of thrombolytic therapy (6.4% vs. 6.1%, *p* = 0.7). In-hospital mortality rates were higher in women than in men [4.55% (80/1757) vs. 2.34% (59/2521), *p* < 0.0001], and neurological deterioration was more frequent in the former than in the latter (9.9% vs. 6.0%, *p* < 0.01). The risk of IICP was significantly higher in patients with AF than in patients without AF [13.2% (101/766) vs. 1.7% (59/3512), *p* < 0.001]. In the patients with LAA, the risk of IICP was not different between male and female patients [3.0% (21/710) for men and 3.5% (15/431) for women, *p* = 0.61]. In patients with CE, the risk of IICP did not differ between male and female patients [11.5% (35/304) for males and 16.0% (48/300) for females, *p* = 0.12]. However, the risk of IICP was significantly higher in CE patients than in LAA patients [13.7% (83/604) for CE and 3.2% (36/1141) for LAA, *p* < 0.001].

**Table 1:** Patient characteristics stratified by sex.

Characteristics	Total (n = 4278)	Women (n = 1757)	Men(n = 2521)	P
Age, mean(SD), years	69.89 (12.12)	72.18 (12.18)	68.29 (11.81)	<0.01
Age (years)				
<65	1348 (31.5%)	430 (24.5%)	918 (36.4%)	<0.01
65–74	1243 (29.1%)	483 (27.5%)	760 (30.1%)	
75–84	1273 (29.8%)	603 (34.3%)	670 (26.6%)	
>85	414 (9.7%)	241 (13.7%)	173 (6.9%)	
Smoking	1825 (42.7%)	39 (2.21%)	1786 (70.8%)	<0.01
Comorbidity				
Diabetes mellitus	1877 (43.9%)	818 (46.6%)	1059 (42.1%)	<0.01
Hypertension	3447 (80.8%)	1488 (84.9%)	1959 (77.9%)	<0.01
Atrial fibrillation	766 (17.9%)	393 (22.4%)	373 (14.8%)	<0.01
Previous stroke	1180 (27.6%)	453 (25.8%)	727 (28.9%)	0.03
Hyperlipidemia	1847 (43.5%)	814 (46.9%)	1033 (41.2%)	<0.01
CHADS2 score				
Median (IQR)	2 (1–3)	2 (1–3)	2 (1–3)	<0.01
Stroke severity (NIHSS)				
<5	1908 (44.6%)	718 (40.9%)	1190 (47.2%)	<0.01
5–15	1673 (39.1%)	673 (38.3%)	1000 (39.7%)	
16–25	444 (10.4%)	219 (12.5%)	225 (8.9%)	
>25	253 (5.9%)	147 (8.4%)	106 (4.2%)	
IICP	160 (3.7%)	81 (4.6%)	79 (3.1%)	0.01
Rt-pa	269(6.3%)	107(6.1%)	162(6.4%)	0.65
Stroke subtype				
LAA	1141 (26.7%)	431 (24.5%)	710 (28.2%)	<0.01
CE	604 (14.1%)	300 (17.1%)	304 (12.1%)	
SVO	1485 (34.7%)	592 (33.7%)	893 (35.4%)	
UN	1039 (24.3%)	428 (24.4%)	611 (24.2%)	
OD	9 (0.2%)	6 (0.3%)	3 (0.1%)	
HT	206 (4.8%)	94 (5.4%)	112 (4.4%)	0.17
ND	327 (7.6%)	174 (9.9%)	152 (6.0%)	<0.01
Fatality	139(3.35%)	80(4.55%)	59 (2.34%)	<0.01
Functional outcome				
Good (NIHSS≦2)	1761(41.2%)	622 (35.4%)	1139 (45.2%)	
Poor (NIHSS>2)	2517(58.8%)	1135 (64.6%)	1382 (54.8%)	<0.01

SD: standard deviation, NIHSS: National Institutes of Health Stroke Score, IICP: increase intracranial pressure, Rt-Pa: thrombolytic therapy with recombined tissue plasminogen, LAA: large-artery atherosclerosis, CE: cardioembolic stroke, SVO: small vessel occlusion, UN: undetermined etiology, OD: other determined etiology, HT: hemorrhagic transformation, ND: neurological deterioration, IQR: interquartile range.

### Sex difference in clinical outcomes

**Fig 1:**
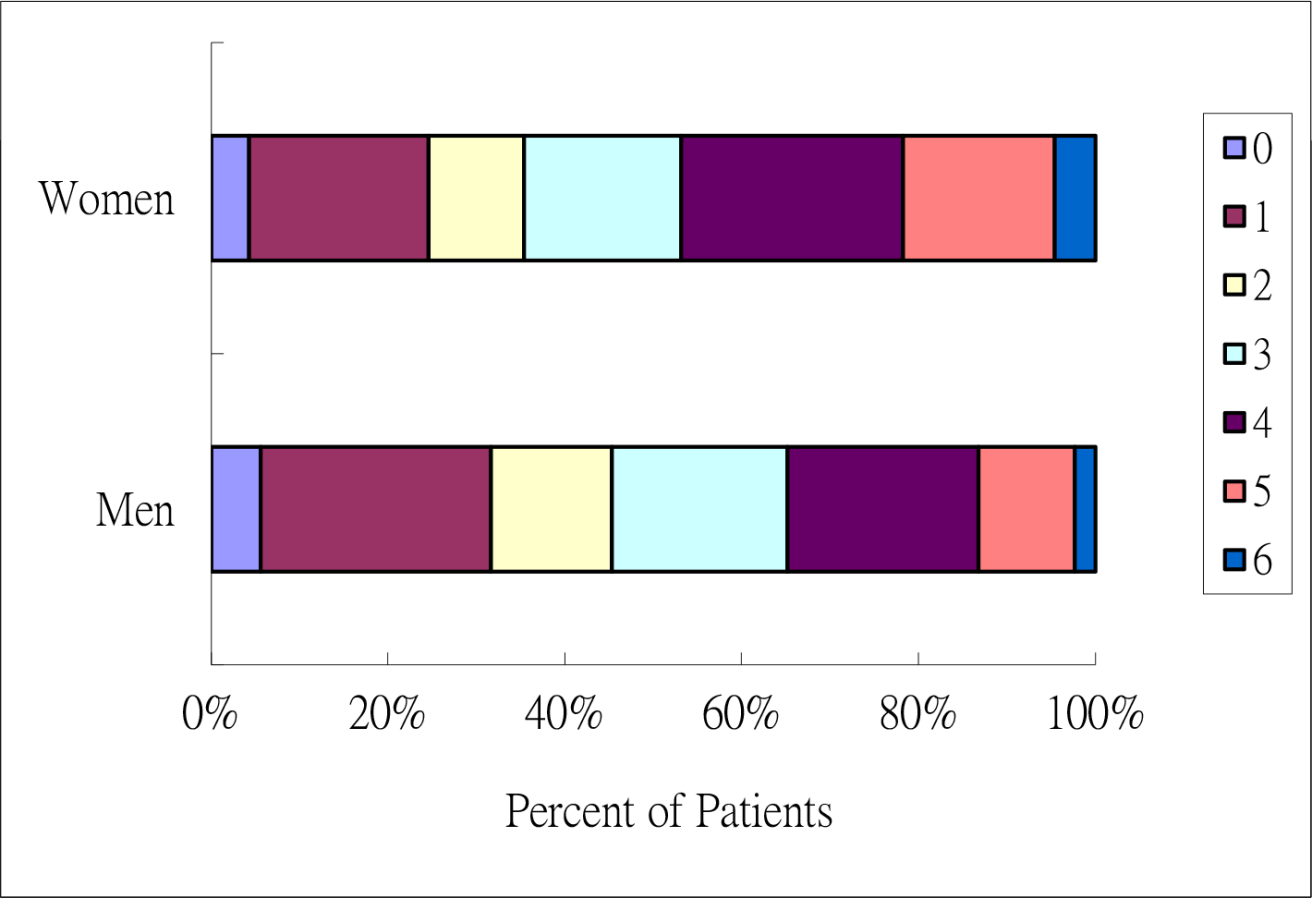
Functional outcome at discharge, according to score on the modified Rankin scale. The figure shows the distribution of Scores on the modified Ranking Scale at discharge. Scores on the Modified Ranking Scale range from 0 to 6, with 0 indicating no symptoms, 1 without clinical disability, 2.mild disability, 3. moderate disability, 4. moderately severe disability, 5. severe disability, 6. death.

**Fig 2:**
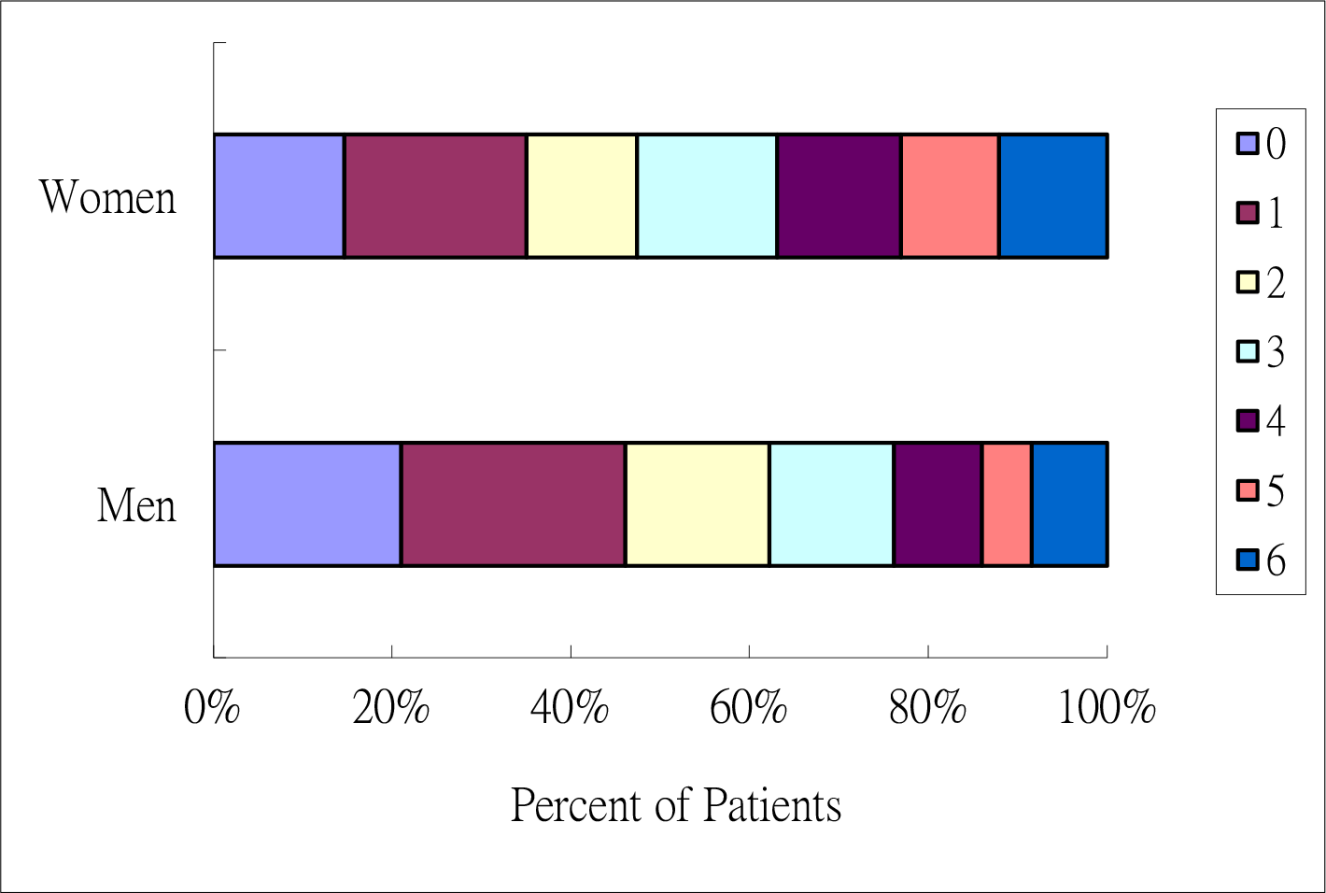
Functional outcome at discharge, according to score on the modified Rankin scale. The figure shows the distribution of Scores on the modified Ranking Scale at 6 months after stroke. Scores on the Modified Ranking Scale range from 0 to 6, with 0 indicating no symptoms, 1 without clinical disability, 2.mild disability, 3. moderate disability, 4. moderately severe disability, 5. severe disability, 6. death.

Univariate analysis revealed that in-hospitality mortality rates were higher in women than in men (P < 0.01). After adjusting for age, stroke severity, and stroke risk factor, in-hospital mortality rates were similar between women and men (odds ratio 1.13, 95% CI 0.66–1.93). Univariate analysis further revealed that an age greater than 75 years increased the risk of in hospital mortality in women but not in men. AF, stroke severity (NIHSS > 15), and IICP increased the risk of in-hospital mortality in both sexes. Cardioembolism stroke increased the risk of in-hospital mortality in women but not in men (Table 2). In patients with AF, female patients had a higher risk of IICP than their male counterparts [15.9% (62/391) in women and 10% (37/369) in men, *p* = 0.02]. At discharge, 41.2% (1761/4278) of the patients showed good outcomes [35.4% (622/1757) of women and 45.2% (1139/2521) of men] (Fig 1, S1 Table). Six months after stroke, 56.1% (1813/3231) of the patients showed good outcomes [47.4% (629/1328) for women and 62.2% (1184/1903) for men, *p <* 0.01] (Fig 2, S2 Table). After adjust age and stroke risk factors, multivariate analysis showed that severe stroke (NIHSS > 15) and IICP increased the risk of in-hospital mortality in both men and women. AF increased the risk of in-hospital mortality in women but not in men. Compared with LAA patients, female CE patients had lower risks of in-hospital mortality but not male CE patients (Table 2). AF increased the risk of stroke severity, ND, HT, and poor outcomes in both sexes. Patients with a history of stroke showed increased risks of severe stroke and poor outcomes, regardless of sex, as well as increased risk of HT in men but not in women. Patients older than 75 years were demonstrated increased risk of ND and poor outcome in both sexes. Old age was also associated with increased stroke severity in women. Diabetes mellitus was associated with increased risk of HT in men and poor outcomes in both men and women. Hypertension was associated with poor outcomes in women but not in men (Table 3).

**Table 2:** Risk factors for in-hospital mortality: Multivariate analysis stratified by sex.

	Total (n = 4278)			Women (n = 1756)			Men (n = 2521)		
Characteristics	% died at discharge	OR (95%CI)	P	% died at discharge	OR (95%CI)	P	% died at discharge	OR (95%CI)	P
Total	3.2			4.6			2.3		
Age group, y									
<65	1.8	1		1.9	1		1.7	1	
65–74	2.6	1.19 (0.61–2.14)	0.59	2.7	1.09 (0.38–3.14)	0.88	2.5	1.28 (0.57–2.89)	0.55
75–84	4.2	1.64 (0.90–2.99)	0.14	6.0	1.80 (0.70–4.61)	0.22	2.5	1.34 (0.58–3.08)	0.50
>85	7.2	1.35 (2.49–7.46)	0.40	9.5	1.28 (0.06–3.56)	0.64	4.0	1.24 (0.42–3.68)	0.70
Smoking									
No	4.1	1		4.6	1		2.8	1	
Yes	2.1	0.94 (0.60–1.48)	0.8	1.7	0.33 (0.04–3.04)	0.30	2.2	1.10 (0.61–2.21)	0.66
Comorbidity									
Diabetes mellitus									
No	3.3	1		4.9	1		2.2	1	
Yes	3.2	0.94 (0.62–1.43)	0.77	4.0	0.85 (0.48–1.50)	0.58	2.5	1.02 (0.54–1.94)	0.94
Hypertension									
No	3.3	1		3.8	1		3.1	1	
Yes	3.2	0.98 (0.58–1.66)	0.94	4.7	1.52 (0.62–3.72)	0.36	2.1	0.73 (0.36–1.45)	0.36
Atrial fibrillation									
No	1.8	1		2.1	1		1.7	1	
Yes	9.7	1.95 (1.16–3.30)	0.01	13.0	2.84 (1.38–5.85)	<0.01	6.2	1.39 (0.63–3.08)	0.42
Stroke history									
No	3.2	1		4.2	1		2.4	1	
Yes	3.4	0.64 (0.40–1.00)	0.05	5.3	0.64 (0.35–1.18)	0.15	2.2	0.59 (0.29–1.17)	0.13
Hyperlipidemia									
No	3.8	1		5.1	1		2.9	1	
Yes	2.2	0.77 (0.50–1.19)	0.23	3.2	0.89 (0.50–1.59)	0.69	1.5	0.60 (0.30–1.22)	0.16
NIHSS									
≦5	0.6	1		0.8	1		0.4	1	
6–15	1.0	1.35 (0.59–3.06)	0.50	1.2	1.20 (0.37–3.87)	0.76	0.8	1.42 (0.44–4.70)	0.55
16–25	9.7	7.79 (3.68–16.48)	<0.01	10.0	6.07 (2.080–17.70)	<0.01	9.3	10.10 (3.39–30.07)	<0.01
>25	27.3	22.24 (10.17–47.52)	<0.01	29.9	17.9 (6.09–52.67)	<0.01	23.6	24.48 (9.46–85.78)	<0.01
IICP									
No	2.0	1		2.8	1		1.5	1	
Yes	35.0	5.94 (3.66–9.62)	<0.01	40.7	4.76 (2.43–9.36)	<0.01	29.1	7.12 (3.48–14.58)	<0.01
Stroke type									
LAA	3.8	1		5.1	1		3.0	1	
CE	7.6	0.46 (0.25–0.86)	0.01	10.0	0.35 (0.15–0.81)	0.01	5.3	0.59 (0.23–1.51)	0.27
SVO	0.2	0.12 (0.03–0.53)	<0.01	0.3	0.23 (0.05–1.06)	0.06	0.1	<0.01 (<0.01-NA)	0.99
UN	4.4	0.91 (0.55–1.50)	0.70	6.1	0.76 (0.37–1.58)	0.46	3.3	1.04 (0.51–2.13)	< .01
OD	11.1	10.14 (1.16–96.89)	0.04	0	0.01< (<0.01-NA)	1	33.3	56.30(4.32–734.59)	<0.01

IICP: increase intracranial pressure, LAA: large-artery atherosclerosis, CE: cardioembolic stroke, SVO: small vessel occlusion, UN: undetermined etiology, OD: other determined etiology, RO: odds ratio, CI: confidence interval.

**Table 3:** Factors affect clinical course in ischemic stroke patients: Multivariate analysis stratified by sex.

Variable (Patient number)	Severity (*p*, 95% CI)		ND (*p*, 95% CI)		HT (*p*, 95% CI)		Poor outcome (*p*, 95% CI)	
	F	M	F	M	F	M	F	M
DM (F:818, M:1059)	0.17 (0.92〜1.55)	0.04 (1.01〜1.67)	0.17 (0.92〜1.55)	0.04 (1.01〜1.67)	0.87 (0.66〜1.61)	0.03 (1.22〜2.76)	<0.01 (0.59〜0.91)	<0.01 (0.68〜0.85)
HTN (F:1488, M:1959)	0.13 (0.53〜1.08)	0.47 (0.66〜1.20)	0.13 (0.53〜1.08)	0.47 (0.66〜1.20)	0.03 (1.06〜4.89)	0.10 (0.43〜1.07)	0.05 (0.56〜1.0)	0.57 (0.77〜1.15)
Lipid (F:814, M:1033)	0.09 (0.61〜1.03)	0.31 (0.68〜1.13)	0.09 (0.01〜1.03)	0.31 (0.68〜1.13)	0.02 (0.37〜0.94)	0.17 (0.88〜1.99)	0.71 (0.83〜1.28)	0.53 (0.80〜1.12)
Af (F:393, M:373)	<0.01 (3.72〜6.34)	<0.01 (3.44〜5.88)	<0.01 (3.72〜6.34)	<0.01 (3.44〜5.88)	<0.01 (3.56〜8.90)	<0.01 (3.98〜8.99)	<0.01 (0.28〜0.52)	<0.01 (0.44〜0.73)
Prior stroke (F:453, M:727)	<0.01 (1.34〜2.31)	0.01 (1.17〜1.94)	<0.01(1.34〜2.31)	0.01 (1.17〜1.94)	0.40 (0.76〜1.92)	0.28 (0.37〜0.94)	<0.01 (0.43〜0.73)	<0.01 (0.36〜0.53)
Age (>75 Y/o) (F:844, M:843)	<0.01 (1.78〜3.07)	0.14 (0.93〜1.56)	<0.01(1.78〜3.07)	0.14 (0.93〜1.56)	0.99 (0.62〜1.59)	0.02 (1.05〜2.40)	<0.01 (0.26〜0.41)	<0.01 (0.35〜0.51)

ND: neurological deterioration, HT: hemorrhagic transformation, CI: confidence interval, DM: diabetes mellitus, HTN: hypertension, Af: atrial fibrillation.

CI: confidence interval.

## Discussion

In this study, stroke onset occurred at an older age in women compared with that in men. The mean ages of stroke onset were 72.18 years for women and 68.29 years for men. While the mean age of stroke onset and the age difference between men and women were consistent with the results in the literature [8, 11–13, 22], our results differed from those of a study conducted in China, which demonstrated stroke onset occurring at an older age in men compared with that in women [14]. A study by Wang et al. showed that the mean age of stroke onset in women was 64 ± 12 years, but our study revealed the mean age of stroke onset in women to be 72.18 ± 12.18 years. The age of stroke onset in men did not differ significantly between these two studies (68.29 ± 11.81 vs. 68 ± 11). According to the TOAST criteria, previous studies report that women are more likely to have cardioembolic stroke and that men are more likely to have large artery atherosclerosis or small vessel disease [9, 23]. Similar to previous studies, in our study, CE accounted for 17.1% of all strokes among women (vs. 12.1% among men). Our findings show that stroke severity was higher in women than in men. The proportion of severe strokes (NIHSS > 15) in women was 20.9% (228/1757) while that in men was 13.1% (185/2521). This result is identical to findings in previous studies [11, 12, 22, 24]. In our study, female patients were more likely to show risk factors of stroke, such as diabetes mellitus, hypertension, hyperlipidemia, and AF (Table 1), than their male counterparts. However, according to Santalucia et al., the presentation of stroke risk factors does not differ significantly between men and women [22]. The same group found that in hospital mortality rates between male and female patients were not significantly different. Further investigation is necessary to determine whether more severe strokes in women are related to the effect of co-morbidities.

In this study, women had higher fatality rates at discharge than men. The background characteristics of male and female patients with ischemic stroke significantly differed. Women were older at stroke onset and suffered from diabetes mellitus, hypertension, hyperlipidemia, and AF more frequently than men. By contrast, men were more likely to have a smoking history. Women also experienced CE more frequently than men, and stroke severity was higher in women than in men. This finding is comparable with those of previous studies [7, 21, 25]. Gender differences in stroke incidence and mortality rates have been reported [7]. In the present study, we found that the risk of in-hospitality mortality was higher in LAA patients that that in CE patients and that AF increased the risk of in hospital mortality in women. This result is inconsistent with the results of Arboix et al [26], who found that in-hospital mortality in patients with AF were significantly higher than that in patients without AF for both CE and atherothrombotic stroke.

Factors related to in-hospital mortality and secondary outcomes differed between men and women. In the present study, the HT rate was not significantly different between men and women, but women were at higher risk of ND. Multivariate analysis revealed that old age increased stroke severity in women but not in men and that stroke history and AF increased stroke severity in both sexes. Differences in stroke severity may be related to the fact that the female stroke patients were older than male stroke patients. Old age and AF increased the risk of ND in both sexes, while diabetes mellitus increased risk of ND in men but not in women. The higher risk of ND observed in women in this study may be related to the fact that our female patients were older and a large proportion of them presented AF and diabetes mellitus. Old age, AF, diabetes mellitus, and stroke history were causes of poor outcomes in both men and women, and hypertension was related to poor outcomes in both sexes. In the present study, poorer outcomes in women than in men were related to the fact that the women were older, suffered from severe stroke, and generally had diabetes mellitus, hypertension, and AF. Differences in-hospital mortality rates between men and women were related to the fact that the women in the study population suffered from more severe strokes and a larger proportion of them had AF and CE compared with men. In consistent with Martin et, al study, our study also showed in the patients with atrial fibrillation, women is less likely CHADS_2_ score 0 or 1 and is associated with higher risk of severe disable or fetal ischemic stroke than men [27]. Yoshida et al study found that in patients with atrial fibrillation, women were older and had larger atrial volume and low left atrial mechanics than men [28]. Our patients with atrial fibrillation, women were older than men and had higher CHADS_2_ score which most related to women were older and more patients had congestive heart failure. In the study, IICP increased the risk of in-hospital mortality (Table 1), and AF increased the risk of IICP. Among patients with AF, females had a higher risk of IICP than males. As in our previous study, AF was found to be a predictor of in-hospital mortality in stroke patients [29]. The result indicates that AF increases in-hospital mortality in women but not in men. This study presents several limitations. First, our work is a one-hospital based study; most of our stroke patients lived in rural areas in central Taiwan, and they may not represent all patients in Taiwan, particularly patients from urban areas. Second, we did not collect information regarding several important pre-stoke conditions, such as pre-stroke dependency and time from stroke onset to hospital admission. Third, the loss follow up rate 1 year after discharge was about 25%. However, the loss follow up rate did not differ between men and women.

## Conclusions

We assessed the factors affecting sex differences in in-hospital mortality and clinical outcomes of stroke patients at discharge and 6 months and found significant differences in terms of stroke severity and stroke risk factors. Women were more likely to have diabetes mellitus, hypertension, AF, and hyperlipidemia than men, and stroke severity was higher in the former than in the latter. While outcomes were poorer in women than in men, this difference can be ascribed to higher stroke severity in women. Our study found that hypertension is an independent factor causing poorer outcomes in women than in men. AF is an independent factor affecting sex differences in hospital mortality in women.

## Supporting information

S1 Table The functional outcome at discharge in 4278 patients. mRS: modified Rankin Scale. 0 indicating no symptoms. 1-without clinical disability. 2-mild disability. 3-moderate disability. 4-moderately severe disability. 5-severe disability. 6-death.
(TIF)

S2 Table The functional outcome at 6 months in 4278 patients. mRS: modified Rankin Scale. 0 indicating no symptoms. 1-without clinical disability. 2-mild disability. 3-moderate disability. 4-moderately severe disability. 5-severe disability. 6-death.
(TIF)
