# Supplementary figures and images for: Sex-related differences in the risk factors for in-hospital mortality and outcomes of ischemic stroke patients in rural areas of Taiwan
Source: PLoS One. 2017 Sep 21;12(9):e0185361. doi: 10.1371/journal.pone.0185361 (PMC5608367; doi:10.1371/journal.pone.0185361)

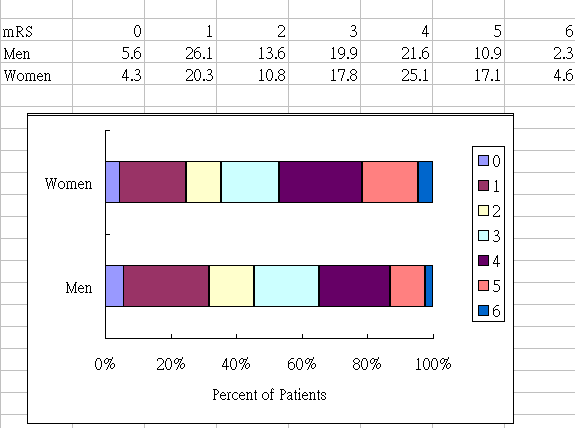

Supplement: S1 Table — mRS: modified Rankin Scale. 0 indicating no symptoms. 1-without clinical disability. 2-mild disability. 3-moderate disability. 4-moderately severe disability. 5-severe disability. 6-death. (TIF) [file pone.0185361.s001.TIF]

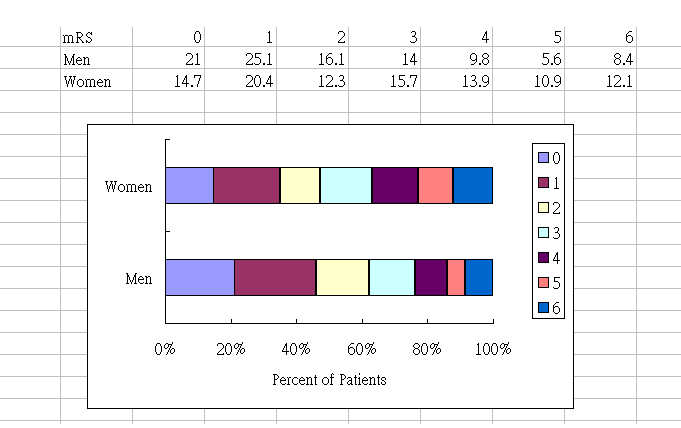

Supplement: S2 Table — mRS: modified Rankin Scale. 0 indicating no symptoms. 1-without clinical disability. 2-mild disability. 3-moderate disability. 4-moderately severe disability. 5-severe disability. 6-death. (TIF) [file pone.0185361.s002.TIF]
